# Resistance to PD-L1/PD-1 Blockade Immunotherapy. A Tumor-Intrinsic or Tumor-Extrinsic Phenomenon?

**DOI:** 10.3389/fphar.2020.00441

**Published:** 2020-04-07

**Authors:** Luisa Chocarro de Erauso, Miren Zuazo, Hugo Arasanz, Ana Bocanegra, Carlos Hernandez, Gonzalo Fernandez, Maria Jesus Garcia-Granda, Ester Blanco, Ruth Vera, Grazyna Kochan, David Escors

**Affiliations:** ^1^ Oncoimmunology Group, Navarrabiomed-UPNA, IdISNA, Pamplona, Spain; ^2^ Department of Medical Oncology, Complejo Hospitalario de Navarra CHN-IdISNA, Pamplona, Spain

**Keywords:** immune checkpoint blockade, programmed cell-death protein 1, programmed cell-death 1 ligand 1, immunotherapy, tumor-intrinsic resistance, tumor-extrinsic resistance, biomarkers

## Abstract

Cancer immunotherapies targeting immune checkpoints such as programmed cell-death protein 1 (PD-1) and its ligand programmed cell-death 1 ligand 1 (PD-L1), are revolutionizing cancer treatment and transforming the practice of medical oncology. However, despite all the recent successes of this type of immunotherapies, most patients are still refractory and present either intrinsic resistance or acquired resistance. Either way, this is a major clinical problem and one of the most significant challenges in oncology. Therefore, the identification of biomarkers to predict clinical responses or for patient stratification by probability of response has become a clinical necessity. However, the mechanisms leading to PD-L1/PD-1 blockade resistance are still poorly understood. A deeper understanding of the basic mechanisms underlying resistance to cancer immunotherapies will provide insight for further development of novel strategies designed to overcome resistance and treatment failure. Here we discuss some of the major molecular mechanisms of resistance to PD-L1/PD-1 immune checkpoint blockade and argue whether tumor intrinsic or extrinsic factors constitute main determinants of response and resistance.

## Introduction

Cancer immunotherapies aim at stimulating the immune system of patients to reactivate its anti-oncogenic activities (Escors, 2014). The most successful anti-cancer immunotherapies are currently those based on immune checkpoint blockade with antibodies (ICIs). Under normal physiologic conditions, immune checkpoints function as regulators of excessive inflammation following T-cell activation, and mechanisms to prevent auto-reactive responses. Unfortunately many cancer cells exploit these T-cell inhibitory mechanisms by up-regulating the expression of immune checkpoint molecules that will bind their ligands on activated T cells leading to their inactivation. It is thought that ICI therapies act primarily on the reactivation of T lymphocytes to exert cytotoxic activities over cancer cells. The emergence of ICI therapies over the last decade has transformed to the core cancer treatments, as they show good efficacies, and less toxicity than conventional chemotherapy or targeted therapies. However, for most cancer types only a subset of all patients effectively respond to these therapies, which is a major clinical, economic, and ethical problem (Topalian et al., 2011; Nishino et al., 2017; Prasad et al., 2017; Kamada et al., 2019; Martins et al., 2019). It is often said that ICI therapies have revolutionized oncology, although their efficacy is still limited. But, what do we mean when we claim that ICI therapies have caused a revolution?

Before the success stories of ipilimumab (Hodi et al., 2008), and before the publication of the results from the first clinical trials of PD-L1/PD-1 blockers (Brahmer et al., 2012; Topalian et al., 2012), immunotherapies were not seriously considered as viable therapeutic options by most oncologists and pharmaceutical companies. Most of their efforts were directed towards the development of small molecule inhibitors for targeted therapies, or novel chemotherapies. And even though targeted therapies showed good efficacies, they were largely limited to patients with tumors harboring the targeted mutations. So, what did ICI treatments truly change? The truly astonishing result is that with only a single drug, objective responses were obtained in a very large number of cancer types largely independent of their ontogeny. Moreover, these drugs are not even directed towards the cancer cell. For example, the anti-PD-1 antibody pembrolizumab has achieved objective responses in cancers as different as melanoma, lung cancer, head and neck, urothelial, gastric cancer, mesothelioma, and Hodgkin lymphoma, among others.

The inhibitory co-receptors that modulate the activation of T cells are generally associated with the T-lymphocyte receptor (TCR) complex at the immunological synapse. These molecules constitute major control points and serve as targets to enhance antitumor immune responses. Some examples expressed in T cells are programmed cell-death protein 1 (PD-1), T-cell immunoglobulin and mucin domain-containing protein 3 (TIM-3), cytotoxic T-lymphocyte antigen 4 (CTLA-4), or lymphocyte-activation gene 3 (LAG-3) (Saito et al., 2010; Chen and Flies, 2013; Esensten et al., 2016; Schildberg et al., 2016; Lichtenegger et al., 2018). Several ICI antibodies targeting CTLA-4 or the PD-L1/PD-1 axis are approved for use by the Food and Drug Administration (FDA) and European Medicines Agency (EMA) for treatment of different cancer types. These antibodies have demonstrated clinical efficacy, with durable clinical responses. Due the success of blockade strategies of CTLA-4 and PD-1 pathways, several antibodies targeting other immune checkpoints are now at different stages of development. Moreover, several combination strategies with ICIs are under evaluation in clinical trials, emerging as new opportunities to enhance anti-tumor immunity (**Table 1**) (Pardoll, 2015).

**Table 1:** Clinical trials targeting the PD-L1/PD-1 axis and combinations.

PD-1/PDL-1 clinical trials		Targets	NCT identifier
**PD-1/PD-L1 monotherapy**		PD-1/PD-L1 axis	NCT03936959, NCT03013101, NCT03167853, NCT03142334, NCT02853344, NCT02702414, NCT02838823, NCT02836795, NCT03010176, NCT03219775, NCT03692442, NCT02358031
**Combination therapies with PD-1/PD-L1 blockade**	**with other immunotherapies**	PD-1/PD-L1 axis and CTLA-4, LAG-3, OX40, TIM-3, GITR, CD20 mAbs, IL2R, IL12, IL7R, IL1B, CD19, CD40, CD38, 41BB	NCT03179007, NCT03615313, NCT03190811, NCT03732547, NCT03970382, NCT03527251, NCT03894215, NCT01968109, NCT02658981, NCT03680508, NCT04198766, NCT04215978
	**with targeted therapies**	PD-1/PD-L1 axis and VEGF/VEGFR, ERK1/2, RAF, AMPK, EGFR, FGFR, MEK, RAF pathways	NCT03851614, NCT04010071, NCT02133742, NCT04152356, NCT03955354, NCT04303741, NCT04014101, NCT03722875, NCT03394287, NCT03359018, NCT02873390, NCT03182816
	**with chemotherapy**	PD-1/PD-L1 axis and direct cancer cell cytotoxicity	NCT03903887, NCT03311789, NCT03737123, NCT04152889, NCT03041181, NCT03515629, NCT03701607, NCT03409614, NCT04225364, NCT02220894, NCT02819518, NCT03221426
	**Other combinations (radiotherapy, chemoradio, multi-way combo, others)**	PD-1/PD-L1 axis and direct cancer cell cytotoxicity	NCT02821182, NCT04017897, NCT03898895, NCT03557411, NCT03984357, NCT03671265, NCT03984357, NCT03619824, NCT03474094, NCT02992912, NCT02434081, NCT02525757

Since 2012, antibodies blocking PD-1/PD-L1 interactions are demonstrating very promising results (Brahmer et al., 2012; Topalian et al., 2012), demonstrating their efficacies and safety. Truly, these results have no precedent in the history of cancer treatments due to their wide range of activities and the durability of responses. To date, six immune checkpoint inhibitors blocking the PD-L1/PD-1 axis are approved by the FDA and the EMA: three PD-1 inhibitors (nivolumab, pembrolizumab, and cemiplimab), and three PD-L1 inhibitors (atezolizumab, durvalumab, and avelumab). Most of them have also been approved by the Chinese National Medical Products Administration (NMPA), and by the Pharmaceuticals and Medical Devices Agency (PMDA) in Japan. Additionally, the NMPA has recently approved the use of four more PD-1 inhibitors (toripalimab, tislelizumab sintilimab, and camrelizumab) in China. These drugs are indicated for the treatment of several cancer types such us melanoma, non–small cell lung cancer (NSCLC), renal cell carcinoma, head and neck squamous cell carcinoma, urothelial carcinoma, microsatellite instability–high colorectal cancer and metastatic cutaneous squamous cell carcinoma.

However, despite these successes the majority of patients in many cancer types do not truly benefit from PD-L1/PD-1 blockade therapies and show resistance, either intrinsic resistance when the treatment directly fails, or acquired resistance where a proportion of responders will also develop resistance. Other patients show some response in the form of stable disease, or acceleration of disease in the form of hyperprogression (Zuazo et al., 2018). Still, the specific mechanisms of resistance and response remain to be elucidated. Therefore, the understanding of the basic mechanistic pathways of resistance and the identification of predictive biomarkers of response have become a clinical necessity. Here, we review the current knowledge on resistance to PD-L1/PD-1 blockade therapies and discuss whether tumor intrinsic or extrinsic factors are the main determinants of response and resistance.

## Programmed Cell Death Protein 1 (PD-1) and Programmed Cell-Death 1 Ligand 1 (PD-L1) Axis

**Figure 1:**
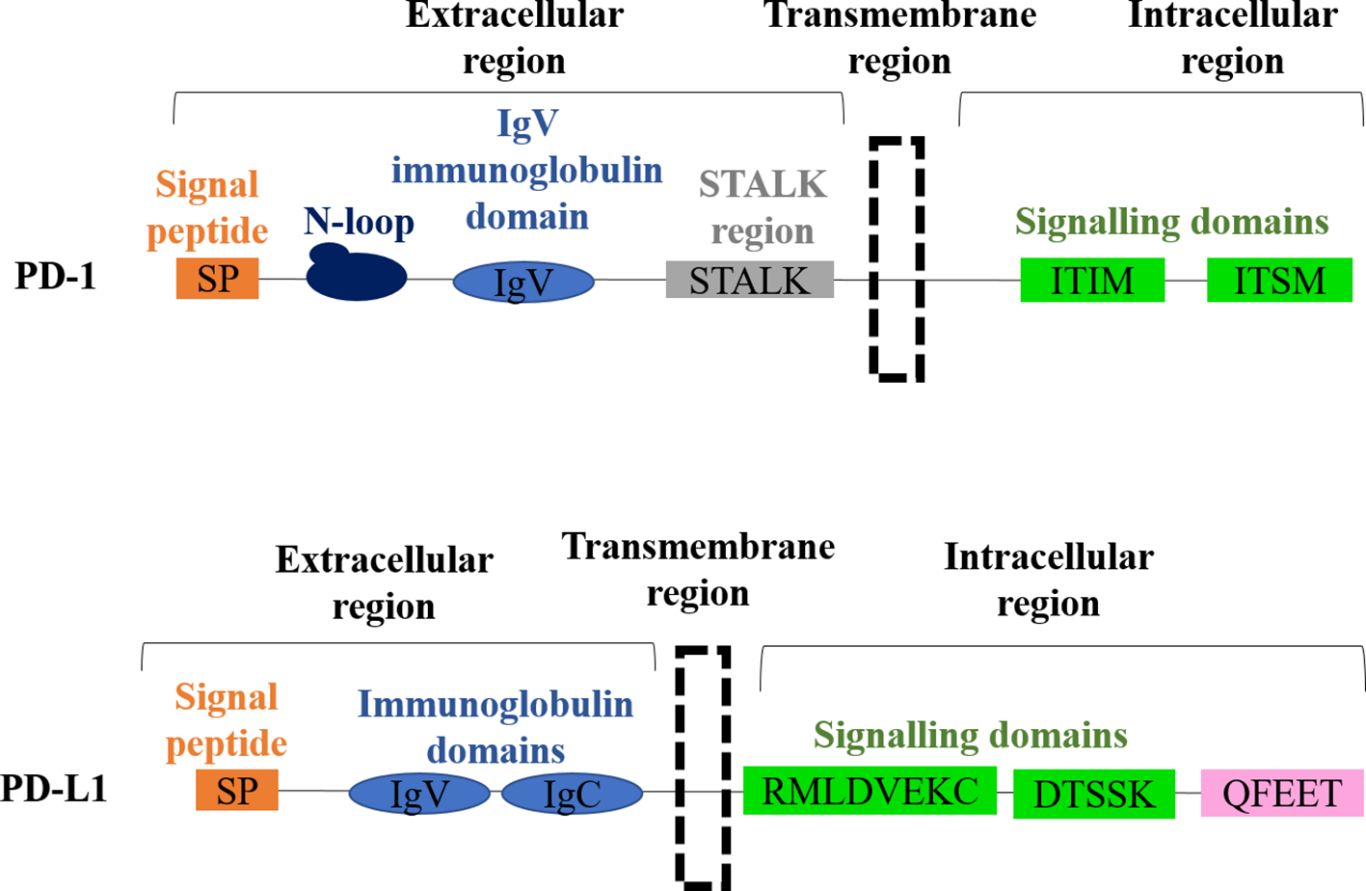
Molecular structures of PD-1 and PD-L1. The domain organization of PD-1 is shown on top, with each domain indicated. The domain organization of PD-L1 is shown below, with each domain indicated.

PD-1 (CD279) is a type 1 transmembrane glycoprotein from the B7-CD28 immunoglobulin superfamily discovered in 1992 for which Prof Honjo received the Nobel Prize (Ishida et al., 1992). This protein is encoded by Pdcd1 gene on the human chromosome 2, and it is composed of a short signal sequence, an extracellular IgV-like domain, a stalk region, a transmembrane domain, and an intracellular cytoplasmatic tail containing the two tyrosine-based signaling motifs; the immunoreceptor tyrosine-based inhibitory motif (ITIM) and the immunoreceptor tyrosine-based switch motif (ITSM) (**Figure 1**). These two motifs contribute to the inhibitory functions of PD-1. PD-1 has two main ligands, PD-L1 (B7-H1, CD274) and PD-L2 (B7-DC, CD273) (Dong et al., 1999; Freeman et al., 2000; Latchman et al., 2001; Tseng et al., 2001) (16–19). PD-L1 is a type I transmembrane protein encoded by the Cd274 gene on the human chromosome 9 discovered in 1999 as an additional member of the B7 family. PD-L1 is composed of a signal sequence, an IgV-like domain, an IgC-like domain, a transmembrane domain, and a highly conserved short intracellular region with intracellular signal transduction capacities (Pascolutti et al., 2016; Gato-Canas et al., 2017; Escors et al., 2018) (**Figure 1**). The intracellular domain presents three highly conserved sequence motifs, two of which are required for regulating interferon-mediated cytotoxicity (RMLDVEKC and DTSSK) (Gato-Canas et al., 2017; Escors et al., 2018). PD-L2 is a type I transmembrane protein encoded by the Pdcd1lg2 gene was discovered in 2001 (Latchman et al., 2001; Tseng et al., 2001) and exhibits a similar molecular oganization than PD-L1.

**Figure 2:**
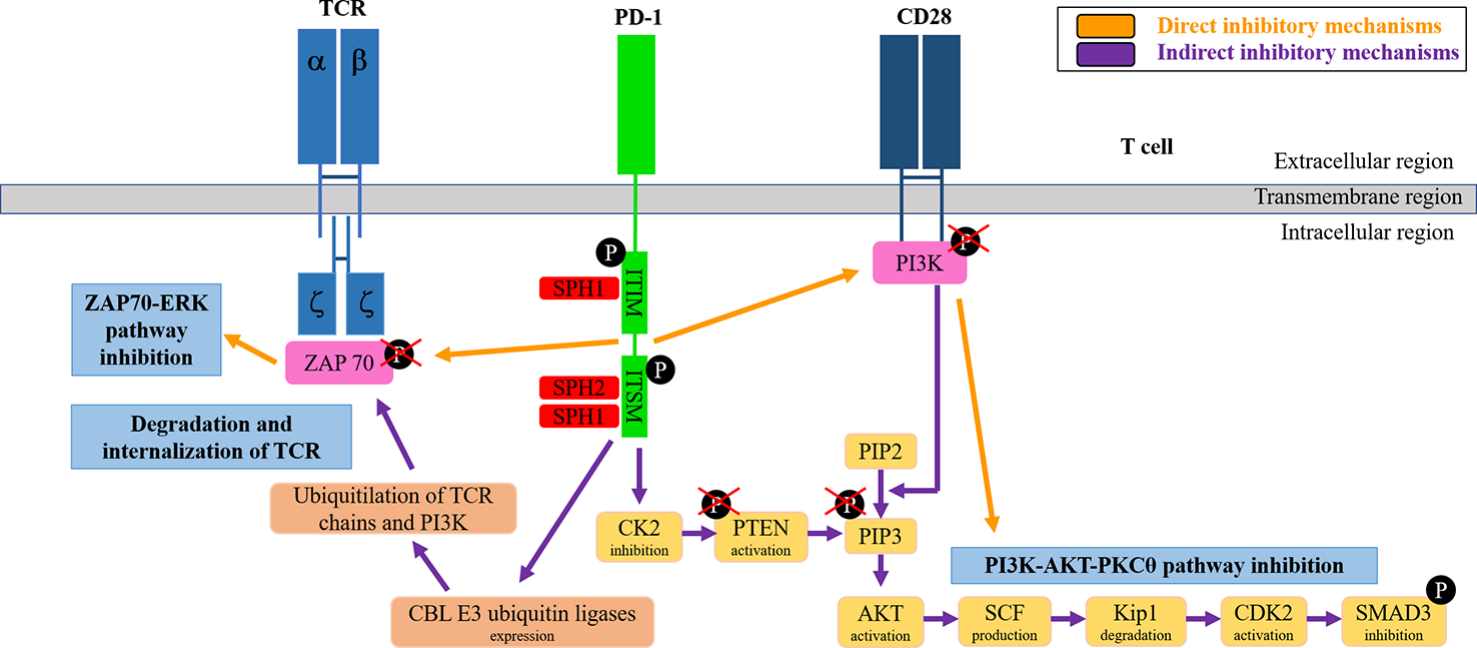
PD-1 signaling pathways in T cells. The figure schematically summarizes the direct and indirect T cell inhibitory signaling mechanisms as indicated.

After engagement with PD-L1, PD-1 inhibits T cell functions through direct and indirect pathways (Arasanz et al., 2017) (**Figure 2**). Direct pathways are dependent on the recruitment of SHP-1 and SHP-2 phosphatases phosphatases to PD-1 ITIM and ITISM motifs following their tyrosine phosphorylation by Lck (Plas et al., 1996; Chemnitz et al., 2004; Sheppard et al., 2004; Hui et al., 2017). SHP phosphatases inhibit ZAP70 and PI3K activities by dephosphorylation, and thus ending the TCR-CD28 signal transduction and its downstream dependent intracellular pathways (ERK and PKCθ). PD-1 also inhibits T cell activities through indirect pathways. After engaged with PD-L1, PD-1 leads to increased expression of CBL E3 ubiquitin ligases, which ubiquitylate components of the TCR leading to its internalization and degradation (Karwacz et al., 2011; Karwacz et al., 2012; Liechtenstein et al., 2014). Also, an indirect pathway of PD-1-dependent inhibition of TCR signal transduction is caused when PD-L1 engages to PD-1 by inhibiting the transcription of CK2 through an unclear mechanism, resulting in de-phosphorylated PTEN that will in turn de-phosphorylate PI3K and terminating in this way downstream pathways (Patsoukis et al., 2013; Arasanz et al., 2017).

In physiological conditions PD-L1/PD-1 interactions keep T cell tolerance toward autoantigens (Latchman et al., 2004). Conversely, in pathological conditions these inhibitory receptors lead to regulation of T-cell effector functions in autoimmunity and infection (Barber et al., 2006; Sharpe et al., 2007). Tumor survival can depend on the PD-L1/PD-1 pathway to attenuate immunogenicity and facilitate resistance to anti-apoptotic stimuli (Hirano et al., 2005; Azuma et al., 2008; Keir et al., 2008; Gato-Canas et al., 2017; Escors et al., 2018). PD-L1 is overexpressed in many tumor types to evade the immune attack and its expression generally (but not always) correlates with progression (Gato-Canas et al., 2017; Escors et al., 2018; Bocanegra et al., 2019; Kattan et al., 2019). PD-1 is expressed in T lymphocytes and interferes with their activation when bound with their ligands PD-L1, inhibiting the effector phase and thus dampening the ability of these T cells to kill cancer cells (Keir et al., 2008; Gato-Canas et al., 2017; Zuazo et al., 2019).

## Mechanisms of Resistance toPD-L1/PD-1 Immunotherapy

PD-L1/PD-1 blockade immunotherapy demonstrates longer duration of responses, and it is better tolerated than traditional therapies. However, despite the recent successes, a large number of patients do not respond to the therapy. This fact indicates intrinsic (or primary) resistance. In addition, a percentage of responder patients end up progressing through mechanisms of acquired resistance. Primary and acquired resistances are important barriers in terms of benefit to the patient (Pitt et al., 2016; Restifo et al., 2016; Sharma et al., 2017; O’Donnell et al., 2019).

Some of the patients treated with PD-L1/PD-1 immunotherapy show hyperprogressive disease, characterized by an unexpected drastic acceleration in tumor growth after the initiation of the therapy with fatal consequences (Champiat et al., 2017; Kato et al., 2017; Saada-Bouzid et al., 2017; Champiat et al., 2018; Ferrara et al., 2018; Zuazo et al., 2018; Kim et al., 2019). Moreover, a certain percentage of responder patients show an apparent progression of neoplastic lesions caused by massive tumor infiltration by immune cells. This response has been termed pseudoprogression, and it is a confounding factor for evaluation of responses by standard techniques such as computerized tomography (Onesti et al., 2019). These variety of atypical responses have prompted the development of immune-related response criteria (irRC) to better characterize the distinct types of responses associated to immunotherapies (Wolchok et al., 2009), in contrast to conventional evaluation criteria by Response Evaluation Criteria in Solid Tumors (RECIST). Nonetheless, the techniques and biomarkers currently integrated in clinical practice are not sufficient to identify responses. A deeper understanding of the mechanisms leading to resistance to PD-L1/PD-1 blockade is required.

In addition, every patient is unique as a result of genetic and clinical backgrounds. Hence, the mechanisms leading to clinical response or resistance are highly complex and might differ not only according to tumor type but also to patient-specific factors. Therefore, the contribution of tumor-cell intrinsic and patient-specific extrinsic factors needs to be elucidated. In the context of immunotherapies, it is unclear which ones are the main determinants of response and resistance.

### Tumor-Intrinsic Factors and Resistance to PD-L1/PD-1 Blockade Therapies

A number of intrinsic characteristics of the patients are prognostic markers. In principle, we will disregard these general characteristics and focus on more specific factors contributing to immunoresistance. Without any doubt, tumor-intrinsic factors definitely contribute to response or progression in immune checkpoint blockade (Sharma et al., 2017; Chowell et al., 2018; Kalbasi and Ribas, 2020).

**Figure 3:**
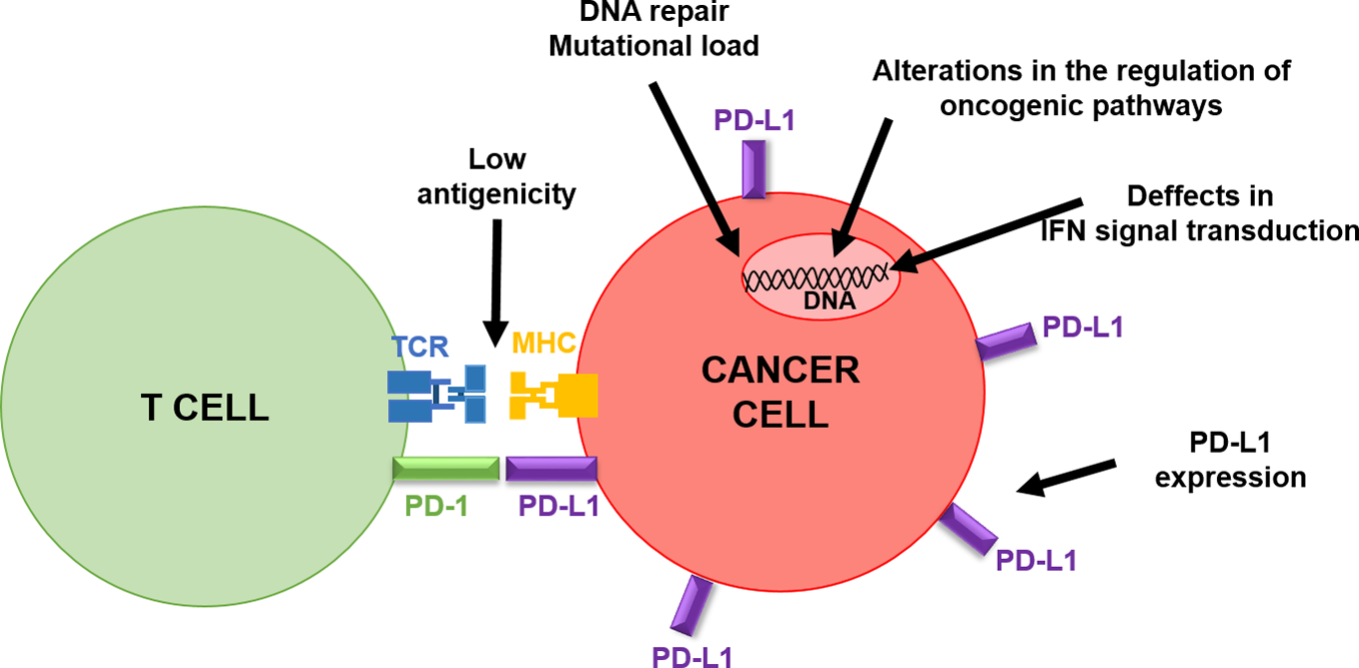
Schematic summary of cancer-intrinsic characteristics influencing clinical responses to PD-L1/PD-1 blockade therapies. The figure depicts the interaction of a T cell with a cancer cell, highlighting cancer cell intrinsic factors that can inactivate T cell activities, as indicated by the arrows.

Tumor-intrinsic factors that contribute to primary and acquired resistance to PD-L1/PD-1 immunotherapy conform a genetic and signaling landscape that prevents immune cell infiltration in the tumor microenvironment (TME) (**Figure 3**). Resistance to PD-1 blockade immunotherapy is often associated with insufficient tumor antigenicity, constitutive PD-L1 expression, defects in IFN signal transduction within cancer cells and alterations in the regulation of oncogenic pathways (Escors, 2014; Sharma et al., 2017).

The loss of tumor antigenicity is a major escape mechanism for many tumor types (Escors, 2014). This is mainly caused by cancer immunoediting, a process by which the immune system exerts a strong and sustained selective pressure over the most immunogenic cancer cell variants (Schreiber et al., 2011). Hence, recognition of tumor-specific antigens by effector T cells is crucial for cancer immunoediting (DuPage et al., 2012). Effector T cells will eliminate the most immunogenic cancer cells and control tumor progression for some time (Restifo et al., 2016; Sharma et al., 2017). However, the less immunogenic cancer cell variants will overgrow and progress. Therefore, tumor immunoediting does constitute a strong mechanism of acquired resistance to immunotherapies. The resulting surviving cancer cells usually show a strong decrease in tumor antigen expression (Matsushita et al., 2012; Escors, 2014), or a down-modulation of molecules involved in antigen presentation such as lack of MHC I or beta-microglobulin expression (Gubin et al., 2014). In this context, ICI therapies will fail simply because no endogenous T cell responses can be raised against these tumors. It has to be noted that immunoediting as a mechanism of immunological escape has been relatively well studied in immunotherapies other than ICIs (Schreiber et al., 2011; Teng et al., 2015; O’Donnell et al., 2019). Therefore, the real extent of the impact of immunoediting over resistance to ICI treatments has not yet been systematically quantified. The detection of less immunogenic variants in samples from patients before the start of immunotherapies may provide the means for adequate patient selection. For instance, characteristics such as genomic instability or epigenetic alterations in pre-existing tumor cell variants, may enable these cancer cells to evade ICI therapies. And these may even facilitate tumor grown, immune evasion, and tumor escape. These escape variants are likely to be naturally selected especially if potent immunostimulatory therapies are applied (Khong and Restifo, 2002). For example, the loss of functional β2 microglobulin from tumor cells, a structural component of the major histocompatibility complex (MHC) 1, confers resistance to tumor-specific CD8 T cells (Restifo et al., 1996). In addition, acquired homeostatic resistance has been described in which tumor cells alter gene expression profiles in response to interactions with the immune system (Pardoll, 2015).

We could include within these mechanisms the adaptive up-regulation of PD-L1 expression as a response to interferons produced during the anti-tumor attack (Garcia-Diaz et al., 2017; Gato-Canas et al., 2017; Escors et al., 2018). Cancer cells with up-regulated PD-L1 would not only inactivate PD-1-expressing T cells, but will also show increased resistance to IFN-mediated apoptosis through reverse signaling by PD-L1 within cancer cells (Gato-Canas et al., 2017; Jalali et al., 2019). It has been known for some time that PD-L1 had intrinsic signaling properties in cancer cells that protected that protected them from a range of apoptotic stimuli, and that its intracellular domain was required for this protection (Azuma et al., 2008). Moreover, PD-L1 was also shown to stimulate cancer cell growth by modulating the activity of AKT/mTOR, autophagy, and glycolysis (Chang et al., 2015; Clark et al., 2016; Gupta et al., 2016). The intracellular part of PD-L1 contains three non-classical signaling motifs; The “RMLD,” “DTSSK,” and “QFEET” motifs (**Figure 1**). The RMLD sequence is required for the anti-apoptotic activities of PD-L1 through the inhibition of STAT3 expression and alternative phosphorylation. The DTSSK motif has regulatory properties, and when it is removed or mutated, PD-L1 molecules exhibit hyperactivated signaling (Gato-Canas et al., 2017). The QFEET motif has been recently shown to be the docking site for the de-ubiquitinase USP22 (Huang et al., 2019).

Inhibition of STAT3 by PD-L1 intrinsic signaling ensures the abrogation of interferon-mediated apoptosis (Gato-Canas et al., 2017), stimulates the inflammasome pathway in cancer cells (Theivanthiran et al., 2020), and directly inhibits PD-L1-positive T cells (Diskin et al., 2020). PD-L1-regulated inflammasome activation triggers a series of signaling cascades that end up with the recruitment of granulocyte myeloid-derived suppressor cells (MDSC) in the tumor environment. This accumulation of MDSCs contribute to resistance to PD-L1/PD-1 blockade strategies. Therefore, PD-L1 expression by cancer cells regulates several pro-carcinogenic mechanisms that can contribute to resistance: First, PD-L1 as an inhibitor of T cell effector activities; second, PD-L1 as an anti-apoptotic shield; and third, PD-L1 as a recruiter of MDSCs into the tumor microenvironment. In agreement with this, it is not surprising that human carcinomas with inactivating mutations in the DTSSK motif of PD-L1 can be selected by immunoediting (Gato-Canas et al., 2017), as these mutations increase the signaling capacities of PD-L1.

Hence, PD-L1 expression in tumors could be considered a tumor-intrinsic factor of resistance. PD-L1 up-regulation in tumor cells is generally associated with tumor progression, proliferation and invasion, antiapoptotic signaling, and T cell inhibitory activities *via* engagement with PD-1 (Escors et al., 2018). PD-L1 expression on tumor cells seems to be sufficient for immune evasion and inhibition of CD8 T cell cytotoxicity (Juneja et al., 2017). Therefore, PD-L1 expression is a recognized biomarker for patient stratification in PD-L1/PD-1 blockade immunotherapy. Some immunohistochemistry assays to quantify PD-L1 expression are currently FDA-approved such as Dako 28-8, Dako 22C3, Ventana SP142, and Ventana SP263. However, the systems of detection are not currently standardized, as different immunochemistry assay and scoring system offer different classifications for tumor PD-L1 status (Arasanz et al., 2018; Bocanegra et al., 2019). Additionally, PD-L1 expression can be highly variable and heterogeneous. Some patients with PD-L1-negative tumors may still benefit from anti-PD-L1/PD-1 therapies as PD-L1 is also expressed by many other cell types including myeloid antigen-presenting cells (Karwacz et al., 2011; Motzer et al., 2015; Horn et al., 2017; Bocanegra et al., 2019). Because of these limitations, PD-L1 expression as a predictive biomarker for responses is still under debate. Nevertheless, the application of radioactively-labeled probes specific for PD-L1 and *in vivo* PET visualization of labeled tumors, and their metastasis is very likely going to solve many of these issues. First, detection of PD-L1 expression levels without the need of obtaining a limited amount of tumor tissue. Second, sensitive detection of “silent” metastases. Third, discrimination of true progression from pseudoprogression, at least for cancers that are PD-L1 positive. So far, several different approaches have been applied in pre-clinical models and in cancer patients. For example, by using PD-L1-specific nanobodies labeled with technetium-99m (Broos et al., 2017), PD-L1-specific cyclic peptides labeled with Gallium (De Silva et al., 2018), and radio-labeled anti-PD-L1 antibodies (Heskamp et al., 2015; Niemeijer et al., 2018).

Several other approaches based on intrinsic tumor characteristics have been established for patient selection. From these, the tumor mutational burden (TMB) has gained popularity as a potential predictive biomarker associated with response to ICI therapies. TMB provides a quantification of the number of mutations per megabase of genomic DNA within the tumor encoding genome. It is thought that “high” TMB tumors may have increased expression of neoantigens and enhanced immunogenicity (Alexandrov et al., 2013; Yuan et al., 2016). Neoantigen load is associated with response and has some predictive value on long-term clinical benefit of PD-L1/PD-1 blockade therapies. The mutational load before the start of immunotherapies seems to be associated to a higher nonsynonymous mutation burden in tumors, higher neoantigen expression, and mutations within the DNA repair pathways (Gubin et al., 2014; Le et al., 2015; Rizvi et al., 2015; Schumacher and Schreiber, 2015). A reflection of this is exemplified by mismatch repair deficiency in cancers, which predicts response to PD-1 blockade for some tumor types such as colon cancer (Le et al., 2015; Le et al., 2017). Therefore, the FDA approved in 2017 the PD-1 inhibitor pembrolizumab for treatment of progressive mismatch-repair deficient solid tumors, consolidating mismatch repair (MMR) defect as a clinically applicable biomarker.

### Tumor-Extrinsic Factors and Resistance to PD-L1/PD-1 Blockade Therapies

**Figure 4:**
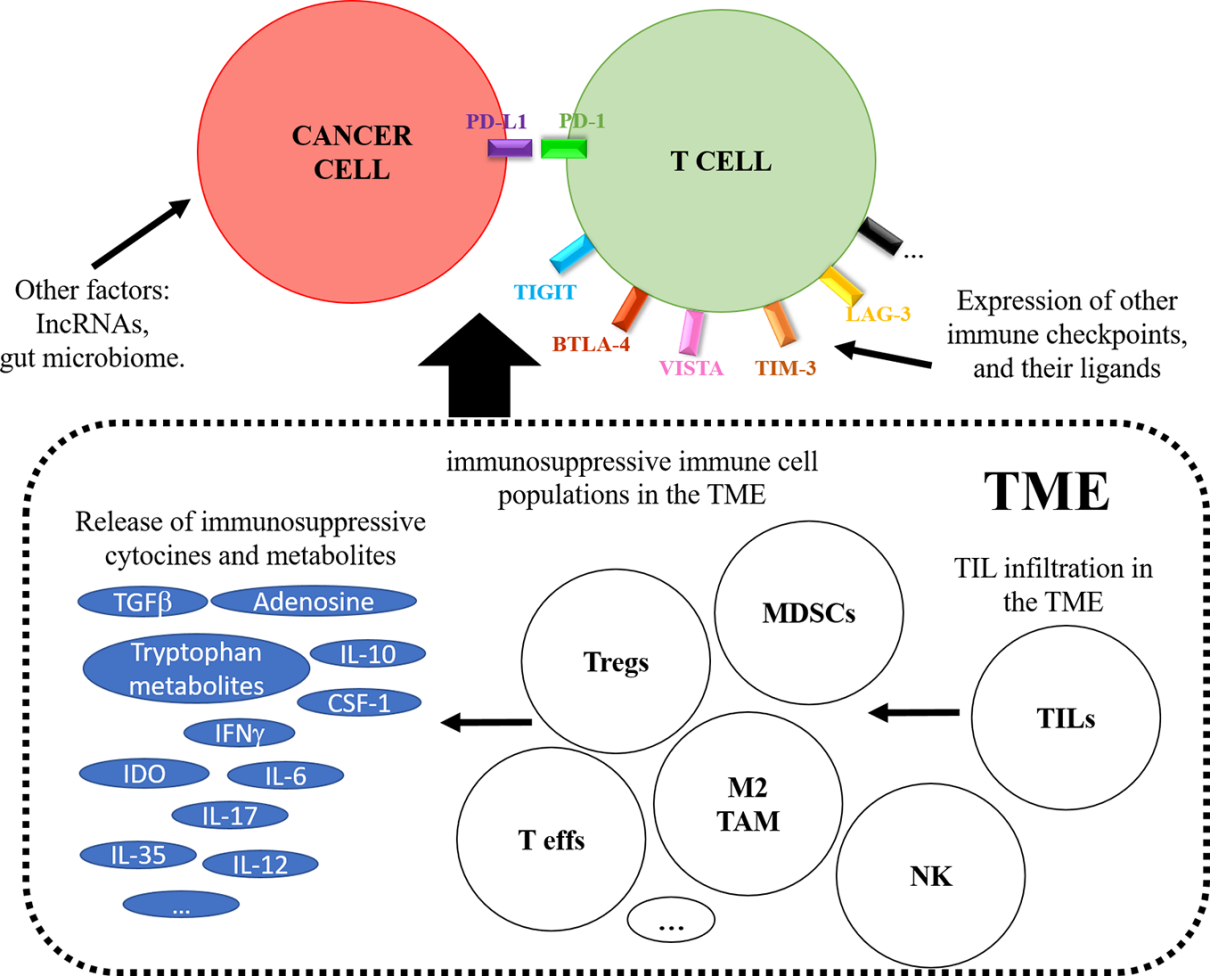
The figure schematically represents tumor-extrinsic mechanisms contributing to response or resistance to PD-L1/PD-1 blockade therapies. The figure depicts on top a T cell interacting with a cancer cell, and the effects caused by the tumor microenvironment (TME) are boxed below. These include the recruitment of immunosuppressive cells as indicated, the expression of immunosuppressive metabolites and the induction of alternative immune checkpoints on the T cell.

ICI immunotherapies differ substantially from conventional therapies in which the target is the immune system. Therefore, it is fair to assume that tumor extrinsic factors linked to the immune system will be associated to response or resistance to ICI therapy. So far, a variety of such factors have been associated to resistance. These include irreversible T cell exhaustion, expression of additional immune checkpoint molecules and their ligands (CTLA-4, TIM-3, LAG-3, TIGIT, VISTA, and BTLA), differentiation and expansion of immunosuppressive cell populations, and release of immunosuppressive cytokines and metabolites both systemically and within the TME (IL-10, IL-6, IL-17, IFNγ, CSF-1, tryptophan metabolites, TGF-β, IDO, increased adenosine production) (**Figure 4**) (Fridman et al., 2017; Sharma et al., 2017; Fares et al., 2019).

One of the oldest prognostic immune biomarkers is the quantification of the type, location, and density of immune cells that infiltrate the TME (O’Donnell et al., 2019). Anti-neoplastic treatments and not only immunotherapies are most efficacious in patients with increased tumor-infiltrating lymphocytes (TILs) in biopsies. This is also true for ICI therapies, and the use of TIL quantification together with PD-L1 tumor positivity is generally associated to good responses (Taube et al., 2012; Bindea et al., 2013). Indeed, there is a positive correlation of TIL infiltration with PD-L1 expression by cancer cells. There are several ways to quantify TIL infiltration, but one of the most successful at least for colon cancer is the so-called “immunoscore” (Galon et al., 2014; Pages et al., 2018; Angell et al., 2020). This biopsy scoring system is a powerful prognostic tool based on the quantification of CD3 and CD8 T lymphocytes on the tumor center and at the tumor invasive margins.

Not surprisingly, TIL infiltration correlates with good prognosis and objective responses to ICI therapies. Oligoclonal TILs are expanded in the tumor tissues of responders to anti-PD-1 blockade. These T cells show enhanced helper T cell type 1 (Th1) cellular immunity (Inoue et al., 2016). Patients can be stratified into four different types according to the characteristics of the TME tumor based on TILs and PD-L1: type I or adaptive immunoresistant (PDL1(+), TIL(+)), type II or immunologically ignorant (PD-L1(-),TIL(-)), type III (PD-L1(+), TIL(-)), and type IV or immune-tolerant (PD-L1(-), TIL(+)) (Teng et al., 2015). This stratification may provide a means for therapy selection. However, other factors contribute to efficacious responses. For instance, the TILs/PD-L1 ratio can be altered according to the expression of oncogene drivers in cancer cells as well as the anatomical location of the neoplastic lesions.

Recent studies demonstrate that ICI therapies do also alter the dynamics and characteristics of systemic immune cell populations. Interestingly, some of these studies highlight the CD28-CD80 costimulation signaling pathway as a major contributor to efficacious responses to ICI (Hui et al., 2017; Zuazo et al., 2019). Indeed, several studies show a key role for IL-12-expressing dendritic cells with cross-presentation capacities for good responses to immunotherapies (Kerkar et al., 2011; Liechtenstein et al., 2014; Goyvaerts et al., 2015; Berraondo et al., 2018; Garris et al., 2018; Etxeberria et al., 2019). These results reinforce the idea that a systemic functional immunity is very likely a required factor for the efficacy of immunotherapies. This was elegantly shown in murine models (Spitzer et al., 2017) as well as in human patients undergoing PD-L1/PD-1 blockade therapies (Kamphorst et al., 2017; Zuazo et al., 2019). A systemic expansion in peripheral blood of a population of CD28+ PD-1+ CD8 T cells was shown in melanoma patients responding to anti-PD-1 therapy (Kamphorst et al., 2017). Patients with non-small cell lung cancer undergoing ICI therapies that presented systemic dysfunctional CD4 T cells that strongly co-expressed PD-1 and LAG-3 failed to respond to therapies (Zuazo et al., 2019). Interestingly, these CD4 T cells did not lose their capacities for multi-cytokine production following *in vitro* stimulation, albeit with a strong Th17-type of responses. These results suggested that these T cells could not be considered exhausted. However, they showed a degree of proliferative dysfunctionality that was indicative of some type of anergy. Importantly, these patient cohorts were enriched in hyperprogressors, suggesting a key role for T cell dysfunctionality in hyperprogressive disease (Zuazo et al., 2019). These results highlighted the up-regulation of LAG-3 as a major escape mechanism to PD-1/PD-L1 monoblockade strategies. Very similar results were obtained in two other independent studies by Kagamu and collaborators, and Julia and collaborators (Julia et al., 2019; Kagamu et al., 2020). In the study by Zuazo et al. responders had a high percentage of highly differentiated CD27^−^ CD28^−^ memory CD4 T cells before starting immunotherapies, and could be used as a predictive biomarker. Similarly, Kagamu et al. identified this population as CD62L^low^ CD4 cells, while Julia et al. described this population as central memory CD4 T cells.

The expansion of immunosuppressive immune cell populations systemically or infiltrating the TME also contributes to extrinsic factors of resistance. Regulatory T cells (Tregs) strongly suppress tumor-specific T cell functions and disrupt effector T cell function. The mechanisms of Treg-mediated immune suppression are varied and include direct cell-to-cell contact and secretion of potent immunosuppressive cytokines such us L-10, IL-35 or TGF-β (Viehl et al., 2006; Sakaguchi et al., 2008; Arce et al., 2011). Some of these cytones will differentiate naïve T cells into inducible Tregs especially in the context of antigen presentation from tolerogenic DCs (Arce et al., 2011). It is increasingly clear the negative impact that the expansion of myeloid-derived suppressor cells have not only in immunotherapy, but also in conventional therapies. Although there is some controversy on their ontogeny and nature, MDSCs englobe a collection of myeloid populations with potent immunosuppressive activities. Tumor infiltrating MDSCs promote angiogenesis, tumor cell invasion, and establish distal metastatic niches (Srivastava et al., 2012; Meyer et al., 2013; Liechtenstein et al., 2014; Dufait et al., 2015; Gato-Canas et al., 2015; Ibanez-Vea et al., 2017). A special case of immunosuppressive myeloid cells constitutes tumor associated macrophages (TAMs). Tumor infiltration with TAMs usually correlates with poor prognosis, particularly with M2 macrophages characterized by high production of immunosuppressive cytokines. Therefore, tumor infiltration with M2 macrophages over M1 macrophages has an impact on tumor angiogenesis, invasion, metastasis, and immunosuppression (Chanmee et al., 2014; Gato et al., 2016; Ibanez-Vea et al., 2018). The recruitment of M2 macrophages seems to lead to immunotherapy resistance, and recent reports in murine models of cancer treated with PD-L1/PD-1 blockade therapies link macrophages with hyperprogressive disease by removing therapeutic antibodies through interactions with their Fc receptors (Lo Russo et al., 2019).

Other more subtle mechanisms may also contribute to resistance. In recent years it has been shown that long non-coding RNAs (lncRNAs) constitute systemic regulators of many biological functions including cancer (Schmitt and Chang, 2016). Interestingly, some immune-related lncRNAs regulate immunosuppressive mechanisms leading to immune evasion and resistance to immunotherapy. Some examples include loss of antigen presentation, PD-L1 overexpression, regulation of T-cell exhaustion, and MDSC and Treg differentiation and expansion (Zhou et al., 2019; Zheng et al., 2019).

Finally, recent metagenomic studies have shown that abnormal gut microbiome affects antitumor immunity, influencing on the response to PD-1-based blockade (74, 75). For example, the abundance of *Bifidobacterium* spp. in the gut microbiome enhances anti-PD-L1 therapy efficacy and improves antitumor immunity by affecting dendritic cells (Sivan et al., 2015). Responders to immunotherapy showed abundant *Bifidobacterium longum* and *adolescentis*, *Collinesella aerofaciens*, *Parabacteiodes merdae*, and *Fecalibacterium* spp. on their microbioma, while non-responders had increased abundance of *Ruminococcus obeum* and *Roseburia intestinalis* (Gopalakrishnan et al., 2018; Matson et al., 2018). A large presence of *Akkermansia muciniphila* and *A. muciniphila* contributes to the immunogenicity of PD-1 blockade, and its abundance was correlated with clinical responses. Fecal microbiota transplantations restore the efficacy of IL-12-dependent anti-PD-1 blockade (Routy et al., 2018). These observations are not restricted to PD-L1/PD-1 blockade, as the presence of *Bacteroides* spp in the gut microbioma was required for anticancer immunity in anti-CTLA-4 therapy (Vetizou et al., 2015).

## Discussion and Conclusions

It is undisputed that ICI therapies are currently leading the way for the development of efficacious anti-neoplastic treatments. Nevertheless, it is yet unclear which mechanisms are driving resistance to ICI treatments and how to tackle them. The relative contribution of tumor cell intrinsic and extrinsic factors to primary, adaptive, and acquired resistance is currently highly confusing. A deeper understanding of the mechanisms underlying the complex immunological pathways in cancer and the molecular mechanisms underlying the PD-L/PD-1 blockade will provide insight into the subject.

Considering all the current evidence, we propose that performing highly detailed systemic immunological profiling is right now a requirement for any study involving ICIs. Not only to identify potential responders, but also to monitor the “real time” performance of ICI therapies by quantifying the dynamic changes of immune cell populations. An increasing number of clinical studies are addressing this particular issue by quantification of the relative abundance of distinct immunological populations in peripheral blood. Nowadays, flow cytometry panels composed of more than 10 markers are routinely used for immunological profiling without the need of setting up CyTOF technologies. In a recent study published by our group, quantification of the relative proportion of highly differentiated CD27^-^ CD28^-^ CD4 T cells before the start of immunotherapies was sufficient to identify a cohort of NSCLC patients with a high probability of response to PD-L1/PD-1 blockers (Zuazo et al., 2019). More specifically, responder patients had high percentages of central and effector memory CD4 T cells. This analysis relied on a panel of 8 markers to stain T cells from a small blood sample by standard flow cytometry. Importantly, our study was validated by the results from two similar studies which used other alternative T cell markers. The first study correlated the high baseline frequency of central memory CD4 T cells with response to immunotherapy in NSCLC and renal cancer patients using flow cytometry (Julia et al., 2019). In the second study, NSCLC patients with high baseline percentages of CD62L^low^ effector CD4 T cells quantified by CyTOF had a high chance of responding to PD-L1/PD-1 blockade (Kagamu et al., 2020). The dynamics and behavior of these CD4 T cell subsets were identical to those from highly-differentiated memory CD4 T cells in our study, strongly suggesting that we were all monitoring the same CD4 T cell subsets but with different markers. Cytotoxicity assays performed with peripheral T cells have also been shown to have predictive capabilities for nivolumab efficacy (Iwahori et al., 2019), as well as the quantification of PD-1+ CD8 T cells in peripheral blood after administration of PD-1 blockers (Kamphorst et al., 2017). Therefore, all these studies including our own demonstrate that simple analytical techniques can be effectively applied in clinical practice for defining an immunological profile based on systemic T cell subsets without the need of obtaining a tumor biopsy sample.

In addition, the dynamic changes of the immune populations in peripheral blood provides invaluable clinical information. Changes in T cell compartments have been recently shown by others and us to correlate with progression and even hyperprogression. The study by Kagamu and collaborators showed that a decrease in peripheral CD62L^low^ CD4 T cells right after therapy correlated with acquired resistance (Kagamu et al., 2020). In our particular NSCLC cohort, a low baseline percentage of memory CD27- CD28- CD4 T cells correlated with intrinsic resistance (Zuazo et al., 2019). Moreover, a sudden increase in highly differentiated CD4 T cells (CD4 T_HD_ burst) following the first cycle of immunotherapy was indicative of hyperprogressive disease (Zuazo et al., 2018; Arasanz et al., 2020). The identification of hyperprogressors is also of the outmost importance, as these patients deteriorate very quickly with fatal outcomes. Hence, we propose that the generation of a “systemic immunological file” containing the relative percentages of at least T cell subsets before and after the first cycle of immunotherapies will provide the means to identify patients according to probabilities of response and provide useful information to the clinician.

Considering the most recent evidence, we do think that an “immunological file” on each patient provides information over immediate responses to immunotherapy. However, cancer cells can select several mutations that interfere with the specific molecular pathways stimulated by ICI therapies. For example, mutations in JAK1, JAK2, and beta2-microglobulin in cancer cells abrogate interferon-mediated apoptosis and prevent PD-L1 up-regulation by interferons (Zaretsky et al., 2016; Garcia-Diaz et al., 2017; Sharma et al., 2017; Shin et al., 2017). Some mutations in the DTSSK domain of PD-L1 present in human carcinomas enhance the capacities of PD-L1 to counteract IFN-cytotoxicity by interfering with STAT3 expression and its alternative phosphorylation (Gato-Canas et al., 2017). Moreover, this inhibition of STAT3 has been recently shown to activate the inflammasome in cancer cells leading to the recruitment of granulocytic MDSCs to the tumor and causing acquired resistance to immunotherapy (Theivanthiran et al., 2020). The molecular characterization of cancer cells, particularly focusing on genetic traits and mutations, will identify patients with high risk of acquired resistance. New generation sequencing is currently on the increase in clinical oncology, with panels that cover the major oncogenic and driver mutations. In ICI therapies, it is likely that new panels covering mutations affecting immunological signaling pathways and immune checkpoints will be of relevance in the near future. Currently, this is an expanding research subject that will surely play a key role in the future oncology.

By a better understanding of the key pathways involved in these processes, we will develop treatments to effectively counteract resistance. The identification of truly predictive and prognostic biomarkers of response is currently a top priority in clinical practice. Some therapeutic strategies to overcome resistance could include the modulation of the TME to increase immunogenicity, overcome T-cell exhaustion, enhance tumor infiltration, and modulate epigenetic regulation. The incorporation of the “immunological file” to be included in the clinical profile of each patient could be a practical example. NSCLC patients with dysfunctional CD4 systemic immunity before starting immunotherapies have intrinsic resistance (Julia et al., 2019; Kamada et al., 2019; Zuazo et al., 2019; ; Kagamu et al., 2020). A closer analysis of these patients uncovered a high co-expression of PD-1 and LAG-3 (Zuazo et al., 2019), TIM-3 up-regulation (Julia et al., 2019), or an expansion of Tregs (Kamada et al., 2019). These patients could therefore be selected on the basis of their “systemic immunological profile” for combination therapies with anti-PD-1/anti-LAG-3, anti-PD-1/anti-TIM-3 or anti-PD-1/anti-CTLA4 antibodies. In addition, minimizing immunological escape and the onset of resistance will be likely achieved by combination therapies with targeted therapies. Other combinations such as with chemotherapy, radiotherapy, CAR-T cells, or the application of additional immune checkpoint blockade agents targeting LAG-3, TIM-3, CSF1R, IDO, GITR, or CD134 could be the key to achieve long-lasting clinical responses.

## Author Contributions

LC conceived the review and wrote the first draft. MZ, HA, AB, CH, GF, MG-G, and EB and RV as contributed author to the final form of the review. GK and DE conceived the review and contributed to the writing of the final version.

## Funding

The Oncoimmunology group is supported by: Asociación Española Contra el Cáncer (AECC, PROYE16001ESCO); Instituto de Salud Carlos III, Spain (FIS project grant PI17/02119); Gobierno de Navarra Biomedicine Project grant (BMED 050-2019); TRANSPOCART (Instituto de Salud Carlos III, project: ICI19/00069); “Precipita” Crowdfunding grant (FECYT); Crowdfunding grant from Sociedad Española de Inmunología (SEI); DE is funded by a Miguel Servet Fellowship (ISC III, CP12/03114, Spain); LC is supported by a DESCARTHES project grant (Industry department, Government of Navarre project grant number: 0011-1411-2019-000058); AB and EB are supported by a European Project Horizon 2020-SC1-BHC-2018-2020 project grant; CH is supported by a Roche-funded grant (stop fuga de cerebros); HA is supported by the Clinico Junior 2019 scholarship from AECC; MZ is supported by a scholarship from Universidad Pública de Navarra; and MG.is supported by a scholarship from the Government of Navarre.

## Conflict of Interest

The authors declare that the research was conducted in the absence of any commercial or financial relationships that could be construed as a potential conflict of interest.
